# The Combined Strategy of Baicalin and Oxacillin Sodium Against Methicillin-Resistant *Staphylococcus aureus*: Biofilm Inhibition, Virulence Attenuation and In Vivo Anti-Infection Efficacy

**DOI:** 10.3390/biology15181639

**Published:** 2026-09-16

**Authors:** Chao Ning, Jiale Zhou, Zhiyun Yu, Yuxuan Yang, Mengna Kang, Zhiyao Dong, Yantong Sun, Xin Meng, Haiyong Guo

**Affiliations:** 1 College of Life Science, Jilin Normal University, Siping 136000, China; 2 School of Pharmaceutical Sciences, Jilin University, Changchun 130021, China

**Keywords:** drug combination, methicillin-resistant *Staphylococcus aureus*, biofilm, virulence factor, peritonitis model

## Abstract

Over the past few years, considerable scholarly interest has been directed toward the synergistic application of natural compounds alongside conventional antibiotics to address infections stemming from multidrug-resistant (MDR) pathogens. The primary objective of this research is to investigate the efficacy of baicalin (BA), an extract obtained from *Scutellaria baicalensis*, when used in conjunction with oxacillin sodium (OXS). Specifically, the study evaluates their combined impact on biofilm formation and toxicity reduction in methicillin-resistant *Staphylococcus aureus* (MRSA) strain USA300. Furthermore, a murine peritonitis model induced by MRSA USA300 was developed to determine the therapeutic potential of this combination therapy against infection. Experimental data indicate that the co-administration of BA and OXS does not induce hemolysis in vitro. In comparison to treatments involving either BA or OXS alone, the combined regimen significantly enhances the accumulation of intracellular reactive oxygen species (ROS) within MRSA USA300. Additionally, this synergistic approach suppresses the production of extracellular polymeric substances (EPSs), decreases the overall protein content within the biofilm matrix, and impairs the metabolic functions of biofilm cells. The investigation also revealed that the synergistic application of BA and OXS intensifies the suppression of key virulence determinants, specifically lipase activity and staphyloxanthin production, while simultaneously downregulating the transcription of *sarA* (a global regulator of virulence). These findings substantiate the anti-virulence efficacy of the BA-OXS combination. In a murine model of peritonitis established using MRSA USA300, the healthy mice group, the MRSA USA300 group, the BA group, the OXS group, the combined group of BA and OXS, and the VAN group were set up, with eight mice in each group. The results showed that the combined therapy significantly mitigated body weight reduction, decreased the circulating counts of inflammatory cells, including leukocytes and lymphocytes, and suppressed the secretion of pro-inflammatory mediators such as TNF-α, IL-6, and IL-1β, thereby demonstrating potent anti-inflammatory properties. Furthermore, the co-administration of BA and OXS reduced bacterial burden in the abdominal organs of infected mice and alleviated associated histopathological injuries. Importantly, the treatment regimen exhibited no hepatorenal toxicity in the peritonitis mice, effectively maintaining normal levels. In the plasma of mice suffering from peritonitis, the concentration of malondialdehyde (MDA), a marker of oxidative stress, was reduced, while the activities of catalase (CAT) and superoxide dismutase (SOD) were elevated. This modulation contributes to anti-infective effects. These findings offer a theoretical foundation for subsequent investigations into the synergistic application of natural compounds and conventional antibiotics against MRSA.

## Simple Summary

Methicillin-resistant *Staphylococcus aureus* (MRSA) infections present significant therapeutic challenges and constitute a major public health hazard. While the synergistic application of phytochemicals alongside conventional antibiotics offers a promising strategy to suppress bacterial growth with a reduced risk of triggering resistance, the precise mechanisms underlying this antibacterial effect and its in vivo efficacy remain poorly understood. Our study demonstrates that co-administration of baicalin and oxacillin sodium facilitates the intracellular buildup of reactive oxygen species (ROS). Furthermore, this combination reduces the levels of extracellular polymeric substances (EPSs) and total protein within biofilms, suppresses the metabolic functions of biofilm cells, and hinders the production of key virulence factors, specifically lipase and staphyloxanthin. These observed anti-biofilm and anti-virulence effects are likely attributable to the downregulation of *sarA* gene expression induced by the baicalin-oxacillin sodium mixture. Building on these findings, we further assessed the in vivo anti-infective potential of this combined therapy. The results indicated that this combined therapy significantly decreased the infiltration of inflammatory cells within the peritoneal cavity of murine models, suppressed the production of pro-inflammatory mediators, and reduced bacterial burdens in tissues. Furthermore, it facilitated the restoration of pathological tissue injuries and exhibited protective properties for both hepatic and renal functions. In conclusion, the synergistic application of baicalin and sodium oxacillin effectively impedes MRSA biofilm development and virulence factor synthesis, thereby attenuating pathogenicity in a mouse model of peritonitis induced by MRSA USA300 strain. These findings validate the anti-infective efficacy of integrating plant-derived compounds with conventional antibiotics, offering novel experimental support for clinical strategies aimed at managing peritonitis caused by drug-resistant pathogens.

## 1. Introduction

As a ubiquitous Gram-positive microorganism, *Staphylococcus aureus* colonizes roughly one-third of the global human population [1,2]. This pathogen exhibits a dual nature, functioning both as a commensal organism and an opportunistic invader. Infectious diseases may arise when host immune defenses are compromised or when the microbial ecosystem becomes dysregulated [3,4]. Beyond its ability to inhabit diverse human biological sites, including cardiac muscle, cartilage, and persistent wounds, *Staphylococcus aureus* can also attach to abiotic surfaces like medical implants, facilitating the development of bacterial biofilms [5,6]. These biofilms represent complex, heterogeneous assemblies wherein polysaccharides, proteins, extracellular enzymes, and bacterial DNA interconnect to create an extracellular polymeric substance matrix. Such an architecture serves as a robust protective shield for the microorganisms trapped inside this structure [7]. Evidence from clinical research indicates that the development of bacterial biofilms serves as a primary driver for the chronic nature, persistence, and frequent recurrence of infectious diseases [8,9]. In particular, the emergence of methicillin-resistant *Staphylococcus aureus* (MRSA) and the resistance mechanisms facilitated by its biofilm represent a severe risk to both human and animal well-being. These factors frequently cause standard antibacterial therapies to fail, restrict the range of viable treatment strategies, and substantially elevate the rates of illness and death among those affected [9].

In contrast to conventional antibiotics, which primarily function by directly eliminating bacteria or suppressing their growth, natural plant-derived extracts offer distinct benefits, including diverse availability, a wide range of antimicrobial efficacy, and novel modes of action. These extracts can demonstrate antibacterial properties without imposing the intense selective pressure that typically drives bacterial resistance, thereby significantly postponing the rise of resistant strains. Consequently, they have emerged as a pivotal area of inquiry in the creation of novel antimicrobial agents and as complementary strategies to boost the effectiveness of traditional antibiotics [10,11,12,13]. Baicalin (BA), a flavonoid compound extracted and refined from the traditional Chinese medicinal plant *Scutellaria baicalensis* [14], has been validated to possess multiple pharmacological effects, including antimicrobial [15], antioxidant [16], antineoplastic [17], and anti-inflammatory [18] activities. This compound holds substantial scientific merit and promising application prospects in the realm of new drug development for antibacterial preparations. Current research has substantiated that the antimicrobial efficacy of BA is not contingent upon a solitary regulatory target; instead, it functions via an intricate network characterized by multi-target and multi-pathway synergistic regulation [19]. Specifically, BA impedes the assembly of bacterial biofilms and compromises their structural integrity. Furthermore, it suppresses the activation of quorum sensing signaling cascades in bacteria, thereby obstructing the production and release of virulence factors associated with pathogenic organisms. Conversely, it is capable of modulating the host’s inflammatory signaling cascades, thereby mitigating tissue damage induced by pathogenic intrusion. Furthermore, it stimulates the immune system and bolsters the host’s capacity to eliminate pathogenic bacteria. Consequently, BA demonstrates not only potent antibacterial properties but also distinct benefits in overcoming bacterial resistance and managing infections caused by resistant strains [19].

The pharmacological properties of BA, particularly its capacity to suppress biofilm development and counteract drug resistance, have been substantiated by numerous investigations. Saifi et al. [20] reported that BA markedly reduces the transcription of essential genes involved in biofilm assembly in *Klebsiella pneumoniae*, thereby effectively reversing the strain’s resistance phenotype mediated by biofilms. In another study, Chen et al. [21] illustrated that pairing BA with ampicillin yields a potent synergistic antibacterial impact on MRSA. This combination improves therapeutic outcomes through multiple mechanisms, including disruption of the bacterial cell membrane, inhibition of cell wall biosynthesis, and modulation of genes associated with drug resistance. Furthermore, Luo et al. demonstrated that combining BA with tobramycin results in a time-dependent improvement in the eradication of *Pseudomonas aeruginosa* biofilms, offering novel perspectives for combination therapies targeting biofilm-related infections.

Despite the growing concern over drug-resistant infections [22], there remains a significant gap in understanding the in vivo anti-infective efficacy and antibacterial inhibition mechanisms of BA when used in conjunction with conventional antibiotics. Our prior research indicated that combining BA with either gentamicin (GM) or oxacillin sodium (OXS) yields synergistic or partially synergistic effects against MRSA USA300 and ATCC 310011 [23]. Detailed analysis revealed that 1/4 minimum inhibitory concentration (MIC) BA can reduce the minimum inhibitory concentration of OXS against MRSA USA300 to 1/4 of the original level [23]. The 1/32 MIC BA can reduce the minimum inhibitory concentration of GM against MRSA USA300 to 1/4 of the original level. The 1/2 MIC BA can reduce the minimum inhibitory concentration of OXS or GM against ATCC 310011 to 1/8 and 1/32 of the original level, respectively. Therefore, this study hypothesizes that BA can enhance the antibacterial and anti-toxicity effects of conventional antibiotics against MRSA by regulating biofilm formation, reactive oxygen species (ROS) accumulation, and the expression of genes related to pathogenicity. To verify this hypothesis, we systematically evaluated the synergistic anti-biofilm effects of BA combined with OXS or GM in vitro against MRSA USA300 and ATCC 310011. Additionally, a mouse peritonitis model induced by MRSA USA300 infection was established to further assess the in vivo efficacy of this combined treatment regimen.

## 2. Materials and Methods

### 2.1. Ethical Statement

Female BALB/c mice were obtained from Liaoning Changsheng Biotechnology Co., Ltd. (Benxi, China). All procedures involving mice adhered to the protocol authorized by the Animal Care and Ethics Committee of Jilin University (Approval No. YD202603062) and complied with the standards outlined in the “Guidelines for the Care and Use of Laboratory Animals”.

### 2.2. Bacterial Strains, Cell Cultures, and Chemicals

The experimental strains, specifically ATCC 310011 and MRSA USA300, were obtained from the College of Life Sciences at Jilin Normal University in Siping, Jilin Province, China, and the University of Minnesota in Minneapolis, MN, USA, respectively. For the experimental controls, sterile water served as the negative control, whereas gentamicin (GM) and vancomycin (VAN) functioned as positive controls. Oxacillin sodium (OXS), GM, VAN, and tributyrin were acquired from Aladdin Company based in Shanghai, China. Baicalin (BA), with an HPLC purity of no less than 98%, was supplied by Solarbio Company in Beijing, China. The kits for extracting total bacterial protein, quantifying protein via the Bradford method, and rapidly extracting bacterial genomic DNA were sourced from BestBio Company (Nanjing, China), Bioss Company (Beijing, China), and Sangon Biotech Company (Shanghai, China), respectively. All ELISA kits utilized for the detection of inflammatory factors (LOT: 202607), as well as biochemical markers indicative of liver and kidney function (LOT:202607), were procured from sinobestbio Company (Shanghai, China), while the ELISA kits utilized for assessing oxidative stress markers (SOD, LOT: BF07161510; MDA, LOT: BF07165289; CAT, LOT: BF07165614) were acquired from Bioss Company (Beijing, China).

### 2.3. Measurement of Hemolytic Activity

To prepare a 1% suspension of red blood cells, healthy rabbit plasma was first centrifuged at 1000× *g* for 10 min to eliminate serum, followed by three washes with PBS and subsequent dilution. This suspension was then co-incubated with the experimental agents—specifically, BA at 1/4 MIC (or 1/2 MIC) and OXS at 1/4 MIC (or 1/8 MIC) either individually or combined, as well as BA at 1/32 MIC (or 1/2 MIC) and GM at 1/4 MIC (or 1/32 MIC) either alone or in mixture—at 37 °C for one hour. Following incubation, the samples were centrifuged again at 1000× *g* for 10 min, after which 100 µL of the resulting supernatant was collected to determine absorbance at 570 nm. PBS served as the negative control, whereas 0.2% Triton X-100 functioned as the positive control. The hemolysis percentage was computed using the equation: [(absorbance of test sample at 570 nm − absorbance of PBS at 570 nm)/(absorbance of Triton X-100 at 570 nm − absorbance of PBS at 570 nm)] × 100%.

### 2.4. ROS Content Detection Experiment

Bacterial cells, initially suspended at a density of 1 × 10^8^ CFU/mL, were harvested by centrifugation at 3000 rpm for a duration of 5 min. Following this, the resulting pellet was rinsed with phosphate-buffered saline (PBS) and subsequently resuspended. The bacterial suspension was then adjusted to a concentration of 2 × 10^6^ CFU/mL through dilution. This prepared suspension was exposed to various treatment conditions, including BA monotherapy, OXS monotherapy, and a combination of BA and OXS, followed by incubation at 37 °C for 24 h. After the incubation period, the cells were washed with PBS, and DCFH-DA probe was introduced to achieve a final concentration of 10 μM. The mixture was then kept in the dark at 37 °C for 1.5 h to allow for probe reaction. Subsequent to another PBS wash, fluorescence intensity was quantified using an excitation wavelength of 488 nm and an emission wavelength of 525 nm (Spark™ multifunctional microplate reader, Tecan (Shanghai) Laboratory Equipment Co., Shanghai, China). In this assay, sterile water served as the negative control, while Rosup was utilized as the positive control. In addition, a cell-free control group (TSB + DCFH-DA) was set up for background signal subtraction. An identical procedural approach was employed to evaluate the impact of the combined BA and GM treatment on intracellular ROS levels in MRSA USA300 and ATCC 310011.

### 2.5. Determination of Extracellular Polysaccharide (EPS) Content Experiment

A bacterial suspension was prepared at a concentration of 1 × 10^6^ CFU/mL and subsequently combined with various drug treatments, including BA monotherapy, OXS monotherapy, and a combination of BA and OXS. The mixtures were incubated for 36 h at 37 °C with shaking at 200 rpm. Following incubation, the samples were centrifuged at 3500× *g* for 20 min at 4 °C, and the resulting supernatant was collected. The remaining pellet was resuspended in 2 mL of 10 mM EDTA solution and subjected to another round of centrifugation under low-temperature conditions at 3500× *g* for 20 min. The supernatants from both centrifugation steps were pooled together, after which cold anhydrous ethanol was added at 2.2 times the total volume. This mixture was then incubated at −20 °C for one hour. Subsequently, the sample was centrifuged again at 3500× *g* for 20 min at 4 °C to collect the precipitate. The precipitate was resuspended in 1 mL of sterile water, followed by the addition of a 5% phenol solution and concentrated sulfuric acid. After allowing the reaction to proceed at room temperature for 5 min, the absorbance was measured at 490 nm. These absorbance values were then calculated using the glucose standard curve equation (y = 0.4749x + 0.0817, R^2^ = 0.992). An identical protocol was employed to evaluate the impact of the BA and GM combination on the extracellular polysaccharide (EPS) levels in MRSA USA300 and ATCC 310011.

### 2.6. Determination of Total Protein Content in Biofilms

A bacterial suspension with a density of 1 × 10^6^ CFU/mL was prepared and subsequently introduced into culture media supplemented with various pharmacological agents, specifically categorized into three experimental sets: BA monotherapy, OXS monotherapy, and a combination of BA and OXS. The cultures were incubated at 37 °C for a duration of 24 h. Following incubation, non-adherent bacteria were removed, and the remaining biofilms were rinsed three times using sterile phosphate-buffered saline (PBS). Total protein was extracted from the biofilms in strict accordance with the protocol provided by the bacterial total protein extraction kit. Subsequently, the concentration of the extracted biofilm protein was quantified utilizing the Bradford assay, as per the instructions of the corresponding protein quantification kit. This identical procedural approach was employed to assess the impact of the combined administration of BA and GM on the total protein levels within the biofilms formed by MRSA USA300 and ATCC 310011.

### 2.7. Determination of Cell Metabolic Activity in Biofilms

A bacterial suspension with a concentration of 1 × 10^6^ CFU/mL was prepared and dispensed into 96-well plates in equal volumes alongside various drug treatments, which included groups treated with BA only, OXS only, and a combination of BA and OXS. The plates were then incubated at 37 °C for a duration of 36 h. Following this period, the supernatant was discarded, and a mixture containing 20 μL of MTT solution (10 mg/mL) and 20 μL of TSB medium was added to each well. The plates were subsequently incubated at 37 °C for an additional 3 h. After removing the supernatant once more, 200 μL of DMSO was introduced, and the plates were allowed to stand at room temperature for 2 min. The optical density (OD) was measured at a wavelength of 570 nm. The metabolic activity of the biofilm cells, expressed as a percentage, was calculated using the formula: (OD of the experimental group at 570 nm/OD of the control group at 570 nm) × 100%. This same procedure was employed to assess the impact of the combined treatment of BA and GM on the metabolic activity of biofilms formed by MRSA USA300 and ATCC 310011.

### 2.8. Measurement of Lipase Activity

A bacterial suspension was prepared at a concentration of 1 × 10^6^ CFU/mL and subsequently combined with various drug treatments, including BA monotherapy, OXS monotherapy, and a combination of BA and OXS. The mixtures were incubated at 37 °C for a duration of 15 h. Following incubation, the samples were centrifuged at 8000× *g* for 10 min to collect the supernatant, which was then sterilized by filtration through a 0.22 μm membrane. An Oxford cup was positioned on an LB agar plate supplemented with 1% tributyrin (solubilized in ethanol), into which 150 μL of the filtered supernatant was introduced. After further incubation at 37 °C for 24 h, the diameter of the resulting clear zone on the agar surface was measured. This identical procedure was employed to evaluate the impact of the combined administration of BA and GM on the lipase activity of MRSA USA300 and ATCC 310011.

### 2.9. Staphyloxanthin Assay

A bacterial suspension was prepared at a concentration of 1 × 10^6^ CFU/mL and subsequently exposed to various drug treatments, including BA monotherapy, OXS monotherapy, and a combination of BA and OXS. Following a 24 h incubation period at 37 °C, the bacterial cells were harvested by centrifugation at 10,000× *g* for 2 min. The resulting pellet was washed twice with PBS and then resuspended in 2 mL of methanol. Ultrasonic disruption was performed using a power setting of 400 W, with cycles consisting of 3 s of sonication followed by a 2 s pause, continuing for a total duration of 5 min. The samples were then centrifuged at 8000 rpm for 10 min to collect the supernatant. This extraction procedure was repeated twice, after which the optical density was measured at 450 nm. An identical protocol was employed to assess the impact of the BA and GM combination on staphyloxanthin levels in MRSA USA300 and ATCC 310011.

### 2.10. Detection of sarA Expression Level of Virulence Genes by qPCR

A bacterial suspension was prepared at a concentration of 1 × 10^6^ CFU/mL and subsequently combined with either sterile water or various drug treatments, including groups treated with BA alone, OXS alone, and a combination of BA and OXS. The mixtures were incubated at 37 °C for a duration of 24 h. Following incubation, a 1 mL aliquot of each sample was subjected to centrifugation at 8000 rpm and 4 °C for 1 min, after which the supernatant was discarded. The resulting pellet was treated with lysozyme (10 mg/mL) and allowed to stand for 10 min. Subsequently, 1 mL of TransZol Up reagent (TransGen Biotech Co., Ltd., Beijing, China) was added, followed by vortexing for 1 min and a 5 min incubation at room temperature. The supernatant was then collected, mixed with 200 μL of chloroform via vortexing for 1 min, and left to stand for 15 min. This mixture underwent low-temperature centrifugation at 12,000× *g* for 5 min, and the upper aqueous phase was retained. An equal volume of isopropanol was added to this supernatant, and the solution was allowed to stand for 10 min before being centrifuged at 12,000× *g* and 4 °C for 15 min. The resulting precipitate was washed with 1 mL of 75% anhydrous ethanol and centrifuged again under the same conditions (12,000× *g*, 4 °C, 15 min). After discarding the supernatant, the pellet was air-dried at room temperature for 5 min. Finally, the RNA was dissolved in 50 μL of DEPC-treated water, and its integrity was assessed using agarose gel electrophoresis. Following electrophoresis, the isolated RNA is converted into complementary DNA (cDNA) utilizing a dedicated reverse transcription kit. The sequence information of *sarA* primers is as follows: R-TCTTGTTAATGCACAACAACGTAA; F-TGTTTGCTTCAGTGATTCGTTT. The primer sequence information of 16S rRNA as the internal reference gene is as follows: R-TGATCCTGGCTCAGGATGA; F-TTCGCTCGACTTGCATGTA. A reaction volume of 20 μL was prepared, comprising 10 μL of 2× SYBR Green PCR Master Mix, 1 μL of both forward and reverse primers, 4 μL of RNase-free dH_2_O, and 4 μL of cDNA that had been diluted 20-fold. The thermal cycling protocol began with an initial denaturation step at 95 °C for 2 min, followed by 40 cycles consisting of denaturation at 95 °C for 15 s, annealing at 55.8 °C for 15 s, and extension at 72 °C for 20 s. To verify the absence of sample contamination, RNase-free dH_2_O served as the negative control in place of cDNA. Following the amplification process, the relative expression levels were quantified using the 2^−(△△Ct)^ method. This same experimental approach was employed to assess how the combined application of BA and GM influenced *sarA* expression in MRSA USA300 and ATCC 310011.

### 2.11. The Effect of BA and OXS Combination on MRSA USA300-Induced Peritonitis in Mice

Following the protocol established by Hertz et al. [24], a peritonitis infection model was constructed. Forty-eight female BALB/c mice, each weighing 20 ± 2 g, were acquired from Liaoning Changsheng Biotechnology Co., Ltd. (Benxi, China). These animals were grouped using a simple randomization method. Random numbers were generated using Excel, and the mice were assigned according to the random sequence to ensure that the baseline body weights of each group were consistent, thereby reducing selection bias. Six groups were set up, with eight mice in each group: a blank control group comprising healthy mice, a negative control group, a BA monotherapy group, an OXS monotherapy group, a combined BA and OXS treatment group, and a positive control group receiving VAN. To induce infection, the mice received an intraperitoneal injection of MRSA USA300 culture at a concentration of 2 × 10^8^ CFU/mL. Treatment intervention began 1 h after infection through intraperitoneal injection, involving the administration of saline (for the negative control), BA (100 mg/kg), OXS (7.8 mg/kg), their combination (100 mg/kg BA+ 7.8 mg/kg OXS), or VAN (25 mg/kg). These treatments were delivered every 12 h over a six-day period, during which fluctuations in body weight were recorded. On the 7th day, the animals underwent light anesthesia using ether to facilitate the collection of blood samples from the orbital sinus. The collected blood was subsequently centrifuged at 3000 rpm for 10 min at 4 °C, after which the resulting supernatant was preserved at −20 °C. Finally, the mice were sacrificed, and their livers and kidneys were harvested. Following the dissection of peritoneal tissues, complete blood counts were conducted utilizing a Genvet VH50 hematology analyzer (Genrui, Shenzhen, China). Concentrations of inflammatory mediators, specifically TNF-α, IL-6, and IL-1β, along with markers for hepatic and renal function (including Cr, BUN, AST, and ALT) and oxidative stress parameters (SOD, CAT, and MDA), were quantified in mouse plasma via ELISA, strictly adhering to the manufacturers’ protocols. Subsequently, tissue samples were weighed, homogenized, and subjected to serial dilution. To assess bacterial burden, 100 μL aliquots were plated on TSA agar and incubated at 37 °C for an overnight period. For histological evaluation, tissue specimens were fixed in 4% paraformaldehyde overnight, embedded in paraffin wax, and sectioned to a thickness of 4 μm. The resulting sections underwent H&E staining and were examined using an optical microscope.

### 2.12. Data Analysis

Sample size was set at n = 8 mice per group, based on relevant published studies and power analysis using effect sizes from our preliminary data [25,26]. This sample size balances statistical requirements, animal welfare and the 3R principle. Data are expressed as the mean plus or minus the standard deviation (mean ± SD), with all statistical evaluations carried out via Origin 9.0 software. To assess differences across multiple groups, a one-way analysis of variance (one-way ANOVA) was employed. When the global test indicated a statistically significant variation among groups (*p* < 0.05), post hoc pairwise comparisons were executed using Tukey’s test, which also served to adjust for multiple testing. Within each measured parameter, bars in the figures marked with distinct letters denote statistically significant differences (*p* < 0.05), while those sharing identical letters indicate no significant disparity.

## 3. Results

### 3.1. Safety Analysis

**Figure 1:**
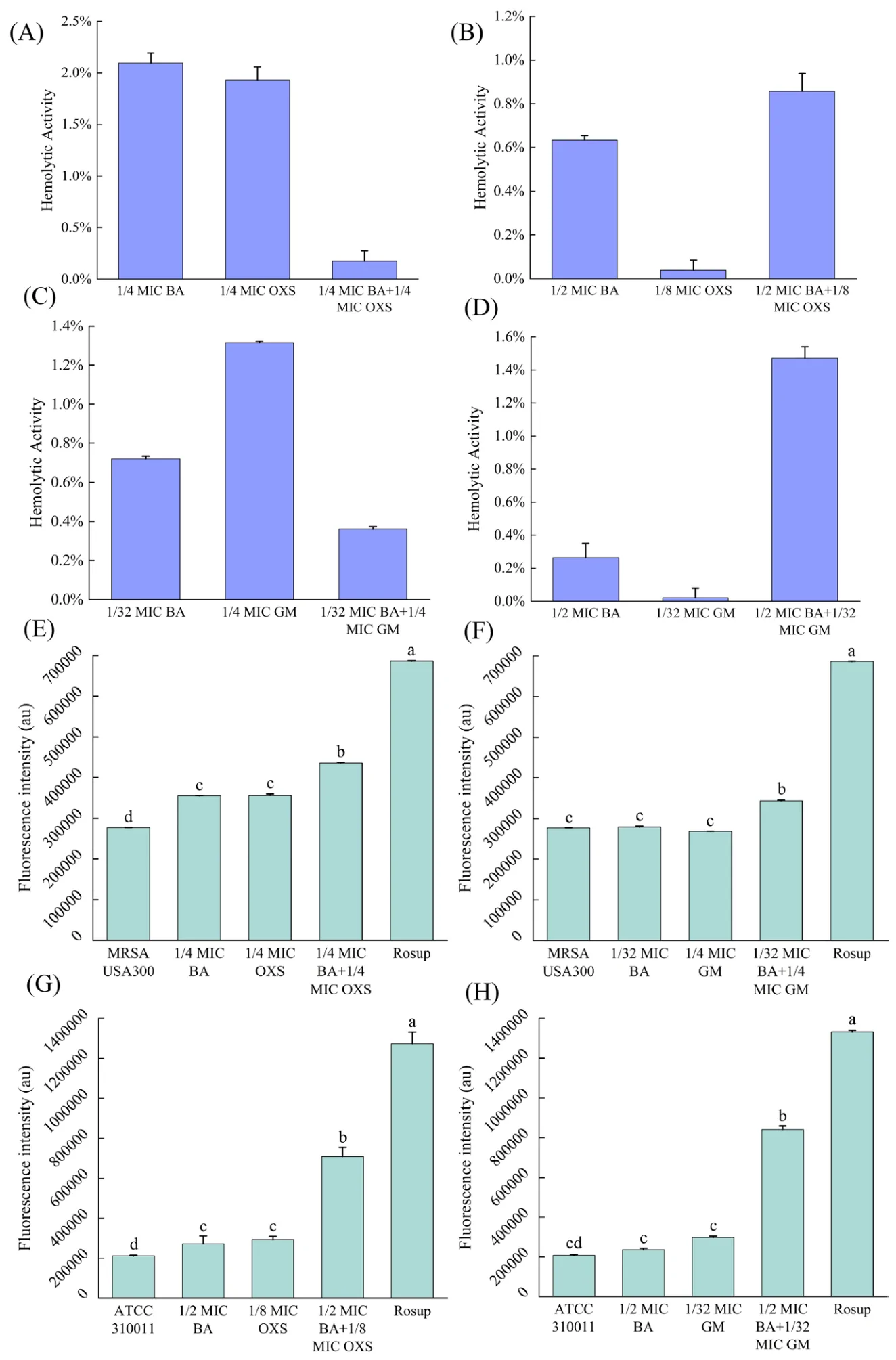
Hemolytic activity of drug combinations and their effects on bacterial ROS levels. (**A**,**B**) Analysis of hemolytic activity for all BA and OXS concentrations used for MRSA USA300 and ATCC 310011; (**C**,**D**) analysis of hemolytic activity for all BA and GM concentrations used for MRSA USA300 and ATCC 310011; (**E**,**F**) effects of BA and OXS or GM combination on ROS levels of MRSA USA300; (**G**,**H**) effects of BA and OXS or GM combination on ROS levels of ATCC 310011.

In this study, the hemolytic potential of the strain was evaluated by examining the effects of all drug combinations used against MRSA USA300 and ATCC 310011 on the integrity of red blood cells. Data analysis revealed that hemolysis rates across all tested drug cohorts remained under the 3% threshold. This finding suggests that the administered concentrations did not induce substantial lysis of red blood cells or significant hemoglobin leakage, thereby confirming a favorable biosafety profile for these treatments (Figure 1A–D).

### 3.2. The Effect of Drug Combination on Bacterial ROS Content

Figure 1E illustrates that untreated MRSA USA300 cells exhibited a comparatively minimal level of intracellular reactive oxygen species (ROS), corresponding to a fluorescence intensity of 276,870 au. Upon individual treatment with either 1/4 MIC BA or 1/4 MIC OXS, the bacteria demonstrated only a marginal increase in ROS accumulation relative to the untreated control group. In contrast, the combined application of these two agents resulted in a substantial elevation of fluorescence intensity to 435,645 au, representing a 1.2-fold increase compared to the group treated solely with 1/4 MIC OXS (Figure 1E). Furthermore, the positive control regimen, consisting of 1/4 MIC GM paired with 1/32 MIC BA, also induced significant intracellular ROS buildup in MRSA USA300. Specifically, the ROS levels in this group were found to be 1.2 and 1.3 times higher than those observed in the untreated MRSA USA300 group and the 1/4 MIC GM group, respectively, as shown in Figure 1F.

For the ATCC 310011 strain, baseline fluorescence remained minimal in the absence of treatment. As illustrated in Figure 1G, administering either 1/2 MIC BA or 1/8 MIC OXS individually failed to produce any notable alteration in fluorescence signals, suggesting that monotherapy with these agents does not influence bacterial intracellular ROS concentrations. In contrast, the co-administration of 1/2 MIC BA and 1/8 MIC OXS triggered a substantial rise in intracellular ROS, yielding a fluorescence intensity of 709,185 au. This value represents a 3.4-fold increase compared to the untreated ATCC 310011 control and a 2.4-fold elevation relative to the group treated solely with 1/8 MIC OXS (Figure 1G). Furthermore, combining 1/32 MIC GM with 1/2 MIC BA resulted in ROS accumulation levels that were 4.1 times higher than those in the ATCC 310011 group and 2.8 times greater than those in the 1/32 MIC GM group (Figure 1H). Collectively, these findings demonstrate that BA markedly potentiates the capacity of both OXS and GM to promote intracellular ROS buildup within *Staphylococcus aureus* cells.

### 3.3. The Effect of Drug Combination on the EPS Content of Bacterial Biofilm Components

**Figure 2:**
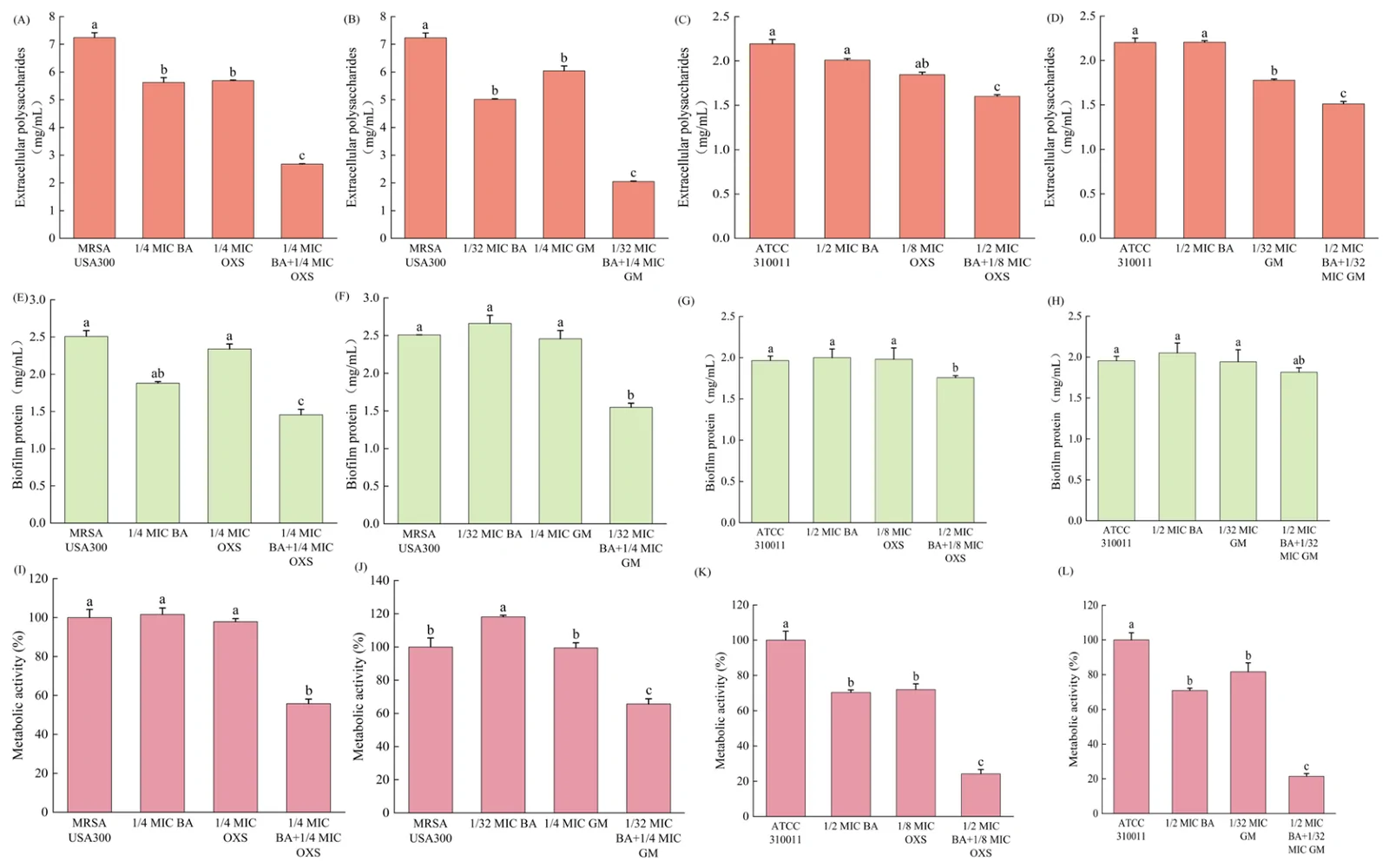
The antibiofilm activity of drug combinations against bacteria. (**A**,**B**) The effects of BA and OXS or GM combined use on the EPS content of the biofilm components of MRSA USA300; (**C**,**D**) the effects of BA and OXS or GM combined use on the EPS content of the biofilm components of ATCC 310011; (**E**,**F**) the effects of BA and OXS or GM combined use on the total protein content of the biofilm of MRSA USA300; (**G**,**H**) the effects of BA and OXS or GM combined use on the total protein content of the biofilm of ATCC 310011; (**I**,**J**) the effects of BA and OXS or GM combined use on the cell metabolic activity of the biofilm of MRSA USA300; (**K**,**L**) the effects of BA and OXS or GM combined use on the cell metabolic activity of the biofilm of ATCC 310011.

As shown in Figure 2A, the EPS content of untreated MRSA USA300 bacteria was 7.24 mg/mL. After being treated with 1/4 MIC BA or 1/4 MIC OXS alone, the EPS content was 5.62 mg/mL and 5.69 mg/mL, respectively; when both were combined, the EPS content significantly decreased to 2.68 mg/mL, which was 63% lower than the MRSA USA300 group and 52.9% lower than the 1/4 MIC OXS group. Similarly, when 1/4 MIC GM was combined with 1/32 MIC BA, the EPS content was 2.15 mg/mL, which was 72.4% lower than the MRSA USA300 group and 66.7% lower than the 1/4 MIC GM group (Figure 2B).

Figure 2C shows that the EPS content of the ATCC 310011 group was 2.19 mg/mL. After being treated with 1/2 MIC BA and 1/8 MIC OXS together, the EPS content significantly decreased to 1.6 mg/mL, which was 26.9% lower than the ATCC 310011 group and 13.5% lower than the 1/8 MIC OXS group. When GM (1/32 MIC) was combined with BA (1/2 MIC), the EPS content of ATCC 310011 was 1.51 mg/mL, which was 31.1% lower than the ATCC 310011 group and 14.7% lower than the 1/32 MIC GM group (Figure 2D). These results indicate that the combination of BA with OXS or GM can significantly inhibit the synthesis of EPS in *Staphylococcus aureus*.

### 3.4. The Effect of Drug Combination on the Total Protein Content of Bacterial Biofilms

Without pharmacological treatment, the overall protein concentration within MRSA USA300 biofilms was measured at 2.51 mg/mL. Upon individual exposure to either BA or OXS at one-quarter of their respective MICs, the protein levels dropped to 1.88 mg/mL and 2.34 mg/mL, respectively. Notably, the concurrent application of 1/4 MIC BA and 1/4 MIC OXS resulted in a more pronounced reduction, bringing the total protein content down to 1.45 mg/mL; this value represents a 38% decrease compared to the group treated solely with OXS (Figure 2E). A comparable trend was observed when GM at 1/4 MIC was paired with BA at 1/32 MIC, yielding a total protein measurement of 1.55 mg/mL for MRSA USA300 biofilms. This combined treatment led to reductions of 38.2% and 37% relative to the untreated MRSA USA300 control and the group receiving only 1/4 MIC GM, respectively (Figure 2F).

Figure 2G illustrates that the baseline total protein concentration in untreated ATCC 310011 biofilms was recorded at 1.96 mg/mL. Following individual exposure to either 1/2 MIC BA or 1/8 MIC OXS, the respective protein levels were measured at 2 mg/mL and 1.98 mg/mL, demonstrating no statistically significant deviation from the untreated control group. In contrast, the synergistic application of BA and OXS led to a marked reduction in biofilm protein content, lowering it to 1.78 mg/mL. A parallel observation was made regarding GM treatment. The 1/32 MIC GM alone resulted in a protein level of 1.94 mg/mL, and its combination with 1/2 MIC BA yielded a value of 1.82 mg/mL, which did not represent a significant decline (Figure 2H). Collectively, these findings suggest that the co-administration of BA and OXS is effective in diminishing the total protein abundance within Staphylococcus aureus biofilms.

### 3.5. The Impact of Drug Combination on Bacterial Metabolic Activity

The solitary application of either BA or OXS at a concentration of 1/4 MIC failed to diminish the metabolic activity of MRSA USA300 biofilm cells relative to the MRSA USA300 group. In contrast, the concurrent administration of these two agents resulted in a metabolic activity level of 55.8% for the biofilm cells. This value represents a reduction of 44.2% when compared to the MRSA USA300 group and a decrease of 43% relative to the group treated with 1/4 MIC OXS alone (Figure 2I). A comparable trend was observed when BA at 1/32 MIC was paired with GM at 1/4 MIC; under these conditions, the metabolic activity of MRSA USA300 biofilm cells was recorded at 65.7% (Figure 2I). Similarly, when 1/32 MIC BA was combined with 1/4 MIC GM, the metabolic activity of MRSA USA300 biofilm cells was 65.7%. Compared to the MRSA USA300 group and the 1/4 MIC GM group used alone, it was 34.3% lower (Figure 2J).

In the case of strain ATCC 310011, exposure to either 1/2 MIC BA or 1/8 MIC OXS individually resulted in biofilm cell metabolic activities of 70.4% and 72%, respectively. However, the concurrent application of both agents reduced the metabolic activity of ATCC 310011 to 24.2%. This value represents a decrease of 75.8% compared to the untreated ATCC 310011 group and a 66.4% reduction relative to the group treated solely with 1/8 MIC OXS (Figure 2K). Likewise, the co-administration of 1/2 MIC BA and 1/32 MIC GM lowered the metabolic activity of ATCC 310011 biofilm cells to 21.4%. This level is 78.6% lower than that of the ATCC 310011 control and 73.8% lower than the group subjected to 1/32 MIC GM alone (Figure 2L). These findings demonstrate that combining BA with either OXS or GM effectively suppresses the metabolic functions of *Staphylococcus aureus* biofilm cells, leading to a substantial decline in bacterial viability.

### 3.6. The Effect of Drug Combination on Bacterial Lipase Activity

**Figure 3:**
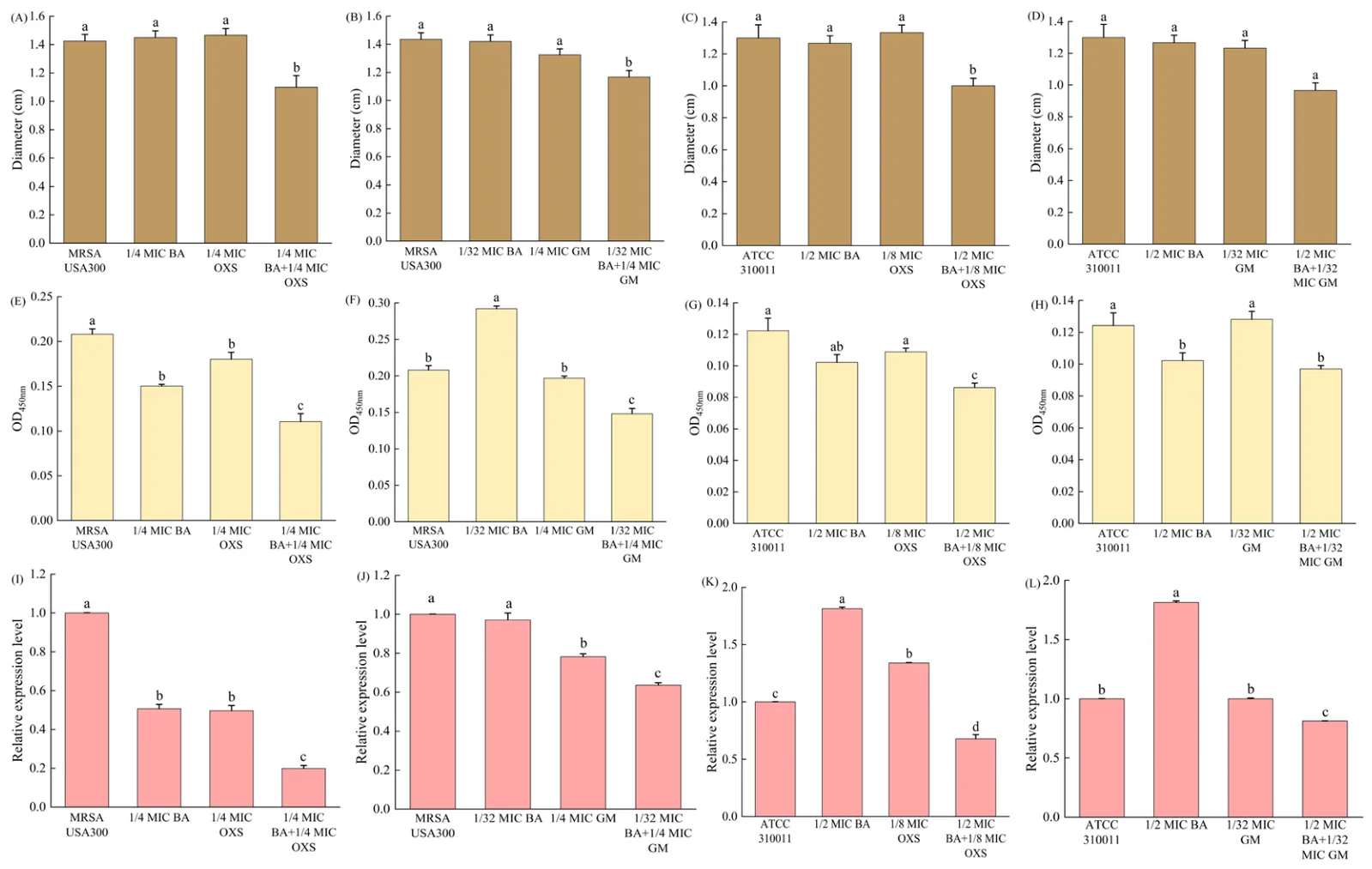
The antibacterial potency effect of drug combination on bacteria. (**A**,**B**) The influence of BA and OXS or GM combined use on the lipase activity of MRSA USA300; (**C**,**D**) the influence of BA and OXS or GM combined use on the lipase activity of ATCC 310011; (**E**,**F**) the influence of BA and OXS or GM combined use on the staphyloxanthin content of MRSA USA300; (**G**,**H**) the influence of BA and OXS or GM combined use on the staphyloxanthin content of ATCC 310011; (**I**,**J**) the influence of BA and OXS or GM combined use on the expression level of *sarA* gene in MRSA USA300; (**K**,**L**) the influence of BA and OXS or GM combined use on the expression level of *sarA* gene in ATCC 310011.

Treatment of MRSA USA300 with either 1/4 MIC BA or 1/4 MIC OXS individually resulted in lipase production zones measuring 1.45 ± 0.1 cm and 1.47 ± 0.1 cm, respectively. However, the concurrent application of 1/4 MIC BA and 1/4 MIC OXS led to a marked reduction in the diameter of these clear zones to 1.1 ± 0.1 cm. This combined treatment yielded diameters that were 23% and 25.2% smaller than those observed in the untreated MRSA USA300 control and the group receiving 1/4 MIC OXS alone (Figure 3A). Similarly, the co-administration of 1/4 MIC GM and 1/32 MIC BA also caused a significant decline in the lipase-induced transparent zone diameter for MRSA USA300, reducing it to 1.17 ± 0.1 cm (Figure 3B). Figure 3C illustrates that BA (1/2 MIC) or OXS (1/8 MIC) as monotherapies resulted in lipase-induced clear zone diameters for strain ATCC 310011 measuring 1.27 ± 0.1 cm and 1.3 ± 0.1 cm, respectively. In contrast, the concurrent administration of 1/2 MIC BA and 1/8 MIC OXS reduced the diameter of these transparent zones to 1 ± 0.1 cm. This measurement represents a 23% decrease compared to either the untreated ATCC 310011 control or the group treated solely with 1/8 MIC OXS. A comparable trend was observed when 1/2 MIC BA was paired with 1/32 MIC GM (Figure 3D). Under this combined regimen, the clear zone diameter shrank to 0.97 ± 0.1 cm, marking reductions of 26.5% and 19.2% relative to the ATCC 310011 control and the 1/32 MIC GM monotherapy group, respectively. These findings suggest that combining BA with either OXS or GM significantly suppresses lipase activity, which in turn diminishes bacterial virulence and the likelihood of infection.

### 3.7. The Influence of Drug Combination on Bacterial Staphyloxanthin Content

For the untreated MRSA USA300 strain, the recorded absorbance at a wavelength of 450 nm stood at 0.21. In comparison, groups subjected to monotherapy with either 1/4 MIC BA or 1/4 MIC OXS exhibited absorbance levels that were below those observed in the control MRSA USA300. A marked reduction was noted when both agents were administered concurrently, with the absorbance dropping to 0.11. This combined treatment resulted in values that were 47.6% lower than the untreated MRSA USA300 group and 38.9% less than the group receiving 1/4 MIC OXS alone (Figure 3E). Furthermore, the combination of 1/32 MIC BA and 1/4 MIC GM yielded an absorbance reading that was 28.6% below the baseline of the untreated MRSA USA300 group and 25% lower than that of the 1/8 MIC GM group (Figure 3F).

Figure 3G illustrates that the untreated ATCC 310011 strain exhibited an absorbance of 0.12 at a wavelength of 450 nm. In contrast, groups subjected to monotherapy with either 1/2 MIC BA or 1/8 MIC OXS demonstrated reduced absorbance levels relative to the ATCC 310011 group. The concurrent application of both agents resulted in an absorbance reading of 0.09, representing a 25% decrease compared to the ATCC 310011 group and an 18.2% reduction relative to the group treated solely with 1/8 MIC OXS. Furthermore, as depicted in Figure 3H, the co-administration of 1/2 MIC BA and 1/32 MIC GM led to a marked suppression of staphyloxanthin production when compared to either the untreated ATCC 310011 cultures or those exposed exclusively to 1/32 MIC GM. Collectively, these findings suggest that combining BA with either OXS or GM effectively impedes the biosynthesis of staphyloxanthin, a key virulence factor in *Staphylococcus aureus*.

### 3.8. The Influence of Drug Combination on the Expression Level of the Bacterial Virulence Gene sarA

Quantitative polymerase chain reaction (qPCR) was employed to assess how *sarA* gene expression levels were influenced by the administration of BA in conjunction with either OXS or GM (Figure 3I–L). Relative to the untreated MRSA USA300 control, individual exposure to 1/4 MIC of BA or OXS resulted in a reduction of *sarA* transcription by 49% and 50%, respectively. Notably, the concurrent application of these agents markedly potentiated the suppression of *sarA* expression, achieving an inhibition rate of 80%, which was 1.6 times higher than that of the group using only 1/4 MIC of OXS (Figure 3I). Furthermore, the co-administration of 1/32 MIC BA and 1/4 MIC GM also demonstrated the capacity to inhibit *sarA* expression in MRSA USA300 strains. This combination yielded a 36% inhibition rate, which exceeded the efficacy of the 1/4 MIC GM monotherapy by 14% (Figure 3J).

In the case of strain ATCC 310011, a distinct reduction in *sarA* gene transcription was observed exclusively within the groups receiving combined drug therapies, as opposed to the untreated ATCC 310011 group (Figure 3K,L). Specifically, when BA at 1/2 MIC was administered alongside OXS at 1/8 MIC, the expression of the *sarA* gene was suppressed by 32.2% (Figure 3K). Furthermore, the co-administration of GM at 1/32 MIC and BA at 1/2 MIC resulted in an 18.8% decrease in *sarA* gene expression levels (Figure 3L). These findings suggest that pairing BA with either OXS or GM could hinder biofilm development, virulence factor production, and metabolic functions in *Staphylococcus aureus*, potentially through a mechanism of downregulating *sarA* gene expression.

### 3.9. The Effects of the Combination of BA and OXS on the Body Weight and the Number of Inflammatory Cells in Mice with Peritonitis Caused by MRSA USA300 Infection

**Figure 4:**
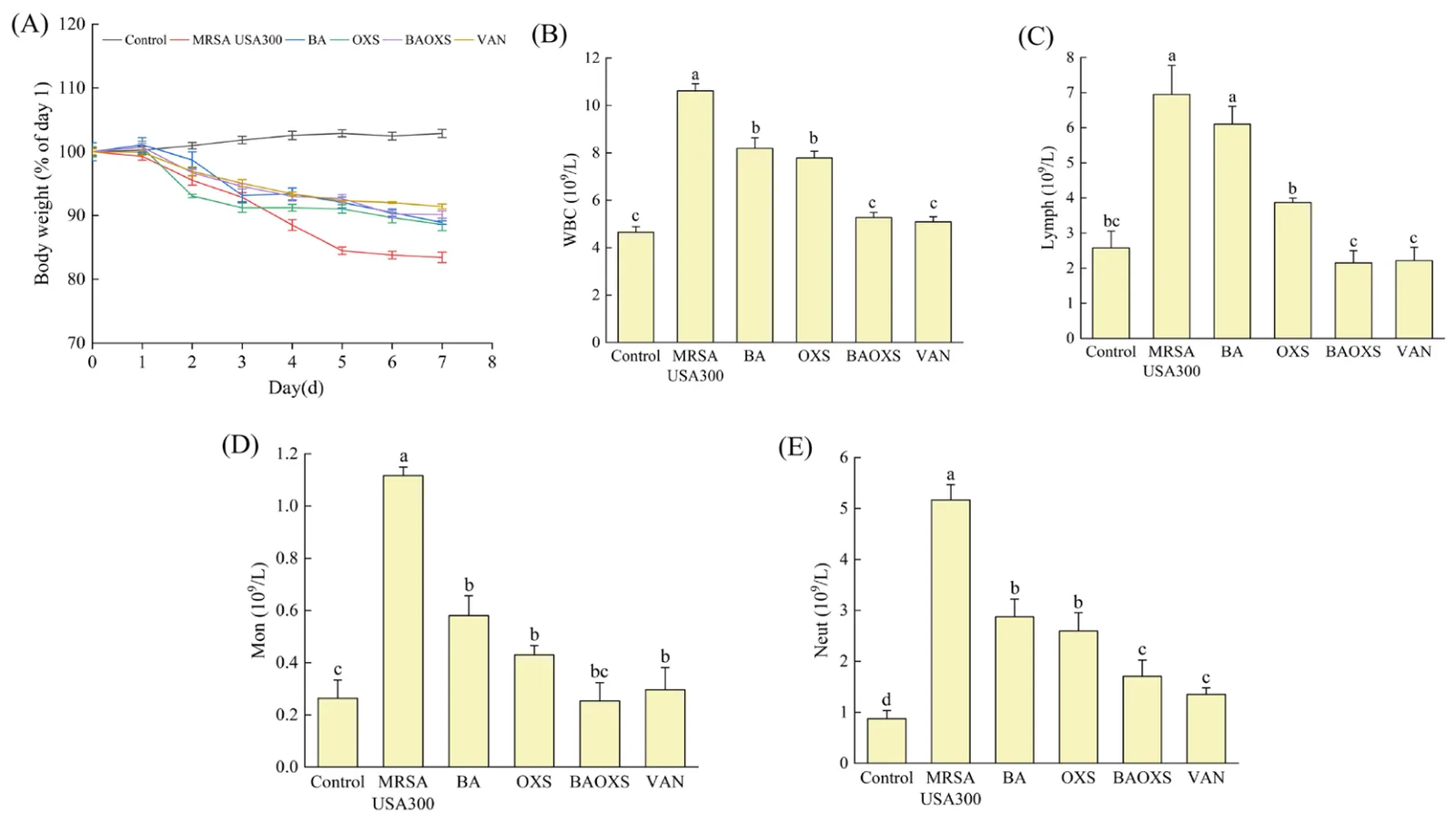
The combined use of BA and OXS on the body weight and inflammatory cell count of mice with MRSA USA300-induced peritonitis. (**A**) The changes in body weight of the six groups were calculated as the percent differences from the initial body weights; (**B**–**E**) the numbers of white blood cells (WBC), lymphocytes (Lymph), monocytes (Mon) and neutrophils (Neut) in the plasma were detected.

By establishing a mouse model of peritonitis caused by MRSA USA300 infection, the in vivo anti-infective efficacy of BA combined with OXS was evaluated. As shown in Figure 4A, the body weight of the control group mice (normal mice) remained stable or even slightly increased within 7 days. In the MRSA USA300 group, the body weight of mice continued to decrease from day 1, indicating that MRSA USA300 infection caused significant weight loss in mice. After intervention with the BA group, the OXS group, the BA and OXS combined group, and the VAN group, the mice also showed weight loss, but the degree of weight loss was significantly less than that of the MRSA USA300 group. This indicates that different drug interventions can, to some extent, alleviate the weight loss caused by infection in mice, and the protective effect of the BA and OXS combined group on mice is better than that of the BA group and the OXS group.

It was also found that, compared with the MRSA USA300 group, the BA group, the OXS group, the BA and OXS (BAOXS) combined group, and the VAN group, the numbers of white blood cells (WBC), lymphocytes (Lymph), monocytes (Mon), and neutrophils (Neut) in the plasma of mice with peritonitis were significantly reduced after intervention (Figure 4B–E). Among them, after intervention with the BA and OXS combined group, compared with the MRSA USA300 group, the counts of white blood cells, lymphocytes, monocytes, and neutrophils in the plasma of mice decreased by 50.4%, 69.1%, 77.3%, and 67%, respectively (Figure 4B–E).

### 3.10. Effects of Combined BA and OXS Treatment on Bacterial Loads and Inflammatory Cytokine Expression in Peritoneal Tissues of MRSA USA300-Infected Peritonitis Mice

**Figure 5:**
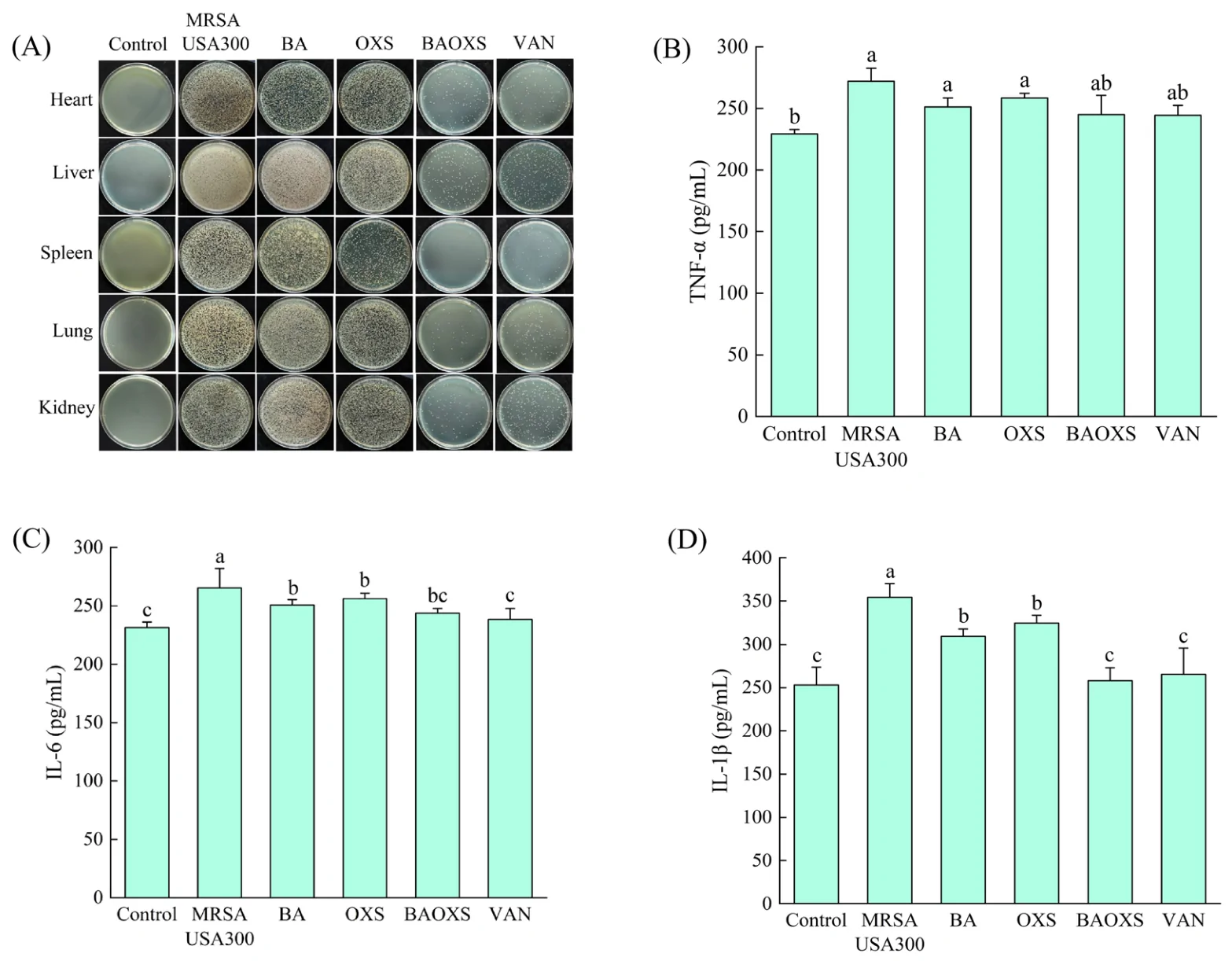
The effects of combined use of BA and OXS on the bacterial load in various tissues (**A**) and inflammatory factors ((**B**): TNF-α; (**C**): IL-6; (**D**): IL-1β) of mice with peritonitis infected by MRSA USA300.

Plate counting assays were employed to assess how the co-administration of BA and OXS influenced bacterial burdens across various organs in murine models of peritonitis. Compared to MRSA USA300, monotherapy with either BA or OXS led to a modest decline in bacterial counts within the heart, liver, spleen, lungs, and kidneys (Figure 5A). In contrast, treatment with the BA-OXS combination or VAN resulted in a substantial reduction in bacterial loads in these same tissues (Figure 5A). Furthermore, an ELISA was utilized to quantify plasma inflammatory markers following therapeutic intervention. Compared to the control group, the MRSA USA300 group exhibited markedly elevated concentrations of pro-inflammatory cytokines, specifically TNF-α, IL-6, and IL-1β (Figure 5B–D). Following administration of the combined BA and OXS, the expression levels of TNF-α, IL-6, and IL-1β were downregulated, with suppression rates recorded at 10%, 8%, and 27%, respectively. When compared to the group receiving the combined treatment of BA and OXS, the individual administrations of either BA or OXS demonstrated a diminished capacity to inhibit the expression of TNF-α, IL-6, and IL-1β in the plasma of mice suffering from peritonitis (Figure 5B–D). These findings suggest that the synergistic application of BA and OXS is capable of significantly decreasing the bacterial burden across various tissues in peritonitis-afflicted mice while simultaneously reducing the plasma concentrations of pro-inflammatory cytokines, thus mitigating the overall inflammatory response.

### 3.11. The Therapeutic Effect of the Combination of BA and OXS on Peritonitis Mice Infected with MRSA USA300

**Figure 6:**
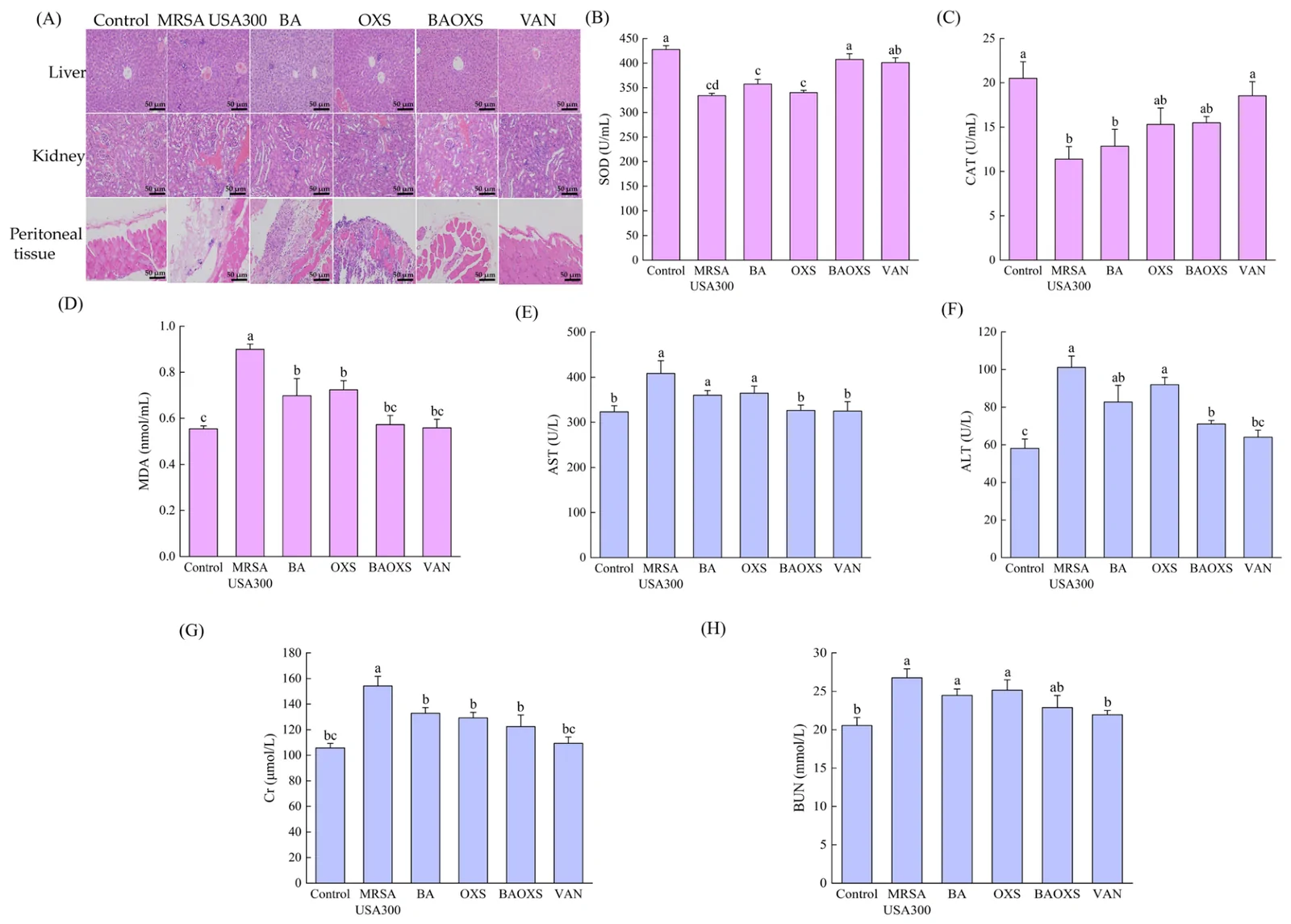
Therapeutic effect of BA and OXS combination on MRSA USA300-infected peritonitis mice. (**A**): Pathological changes were analyzed by hematoxylin-eosin staining. Magnification: ×200. (**B**–**D**) The expression levels of oxidative stress factors MDA, CAT, and SOD in plasma of peritonitis mice after BA and OXS combination intervention were detected. (**E**–**H**) The expression levels of AST, ALT, Cr, and BUN in plasma of peritonitis mice after BA and OXS combination intervention were detected.

Through HE staining, it was found that the liver, kidney and peritoneal tissues of the control group were structurally intact, with normal cell morphology, and there was no obvious degeneration, necrosis or inflammatory cell infiltration. In the MRSA USA300 group, significant tissue damage occurred, manifested as dilatation of renal tubules and epithelial shedding in the kidneys, a small amount of fibrous hyperplasia in the interstitium accompanied by inflammatory infiltration; in the liver, there was loose edema of liver cells and obvious inflammatory cell infiltration; in the peritoneum, a large number of muscle fibers necrotized and dissolved, accompanied by varying degrees of inflammatory cell infiltration (Figure 6A). The BA group and the OXS group still showed moderate liver and kidney damage and necrosis of peritoneal muscle fibers, with limited improvement in tissue pathology (Figure 6A). The BAOXS group exhibited a significant tissue-protective effect, with only slight renal tubular dilation and loose epithelium in the kidneys, no obvious interstitial fibrosis and inflammatory infiltration; in the liver, only a small amount of liver cell edema was observed, and no significant inflammatory cell aggregation was found; the peritoneal muscle fibers were arranged regularly, without muscle fiber necrosis and a large amount of inflammatory cell infiltration (Figure 6A). This indicates that the BAOXS combination can effectively alleviate the pathological damage of liver, kidney and peritoneal tissues mediated by MRSA infection, and has a good protective effect on multiple organ tissue damage caused by infection (Figure 6A). The VAN group only showed mild pathological changes in the liver, kidney and peritoneum (Figure 6A). Subsequently, an ELISA was used to detect the effects of the combination of BA and OXS on the levels of oxidative stress factors and liver and kidney function damage markers in the plasma of mice with peritonitis. Compared to the control, the MRSA USA300 group demonstrated pronounced oxidative injury, characterized by a marked reduction in plasma superoxide dismutase (SOD) and catalase (CAT) activities alongside elevated malondialdehyde (MDA)concentrations (Figure 6B–D). Following administration of BA or OXS, either alone or in combination, SOD and CAT enzymatic functions were recovered, while MDA levels were suppressed (Figure 6B–D). These findings suggest that therapeutic interventions involving BA or OXS, whether applied singly or jointly, effectively mitigate the oxidative stress damage induced by MRSA USA300 (Figure 6B–D). The safety profile of the combined administration of BA and OXS was assessed by monitoring hepatic and renal function indicators in murine models. Relative to the control, mice infected with MRSA USA300 exhibited markedly increased levels of AST, ALT, Cr, and BUN, which suggests that the bacterial infection induced substantial damage to both liver and kidney tissues (Figure 6E–H). Upon treatment with either BA or OXS alone in mice suffering from peritonitis, a decline in these biochemical markers was observed, implying that monotherapy contributed to a partial restoration of impaired hepatic and renal functions (Figure 6E–H). Notably, the concurrent application of BA and OXS resulted in a more pronounced decrease in AST, ALT, Cr, and BUN levels. In particular, the concentrations of AST and BUN were restored to values nearly identical to those recorded in the control group (Figure 6E–H). Consequently, the in vivo safety profile associated with the co-administration of BA and OXS is favorable.

## 4. Discussion

Methicillin-resistant *Staphylococcus aureus* (MRSA) demonstrates resistance to a broad spectrum of antimicrobial agents, including β-lactams, aminoglycosides, quinolones, and macrolides [27]. Given the substantial morbidity and mortality rates associated with infections caused by this pathogen, there is an imperative need to develop novel therapeutic approaches [28]. At present, numerous research groups are focusing on investigating the synergistic application of plant-derived extracts alongside conventional antibiotics to identify promising alternative strategies for combating infections [29,30,31,32,33]. Baicalin (BA) has been confirmed to possess antibacterial properties, and its combination with antibiotics often results in additive or synergistic effects, suggesting that BA could facilitate a reduction in antibiotic dosage [19]. However, the underlying mechanisms governing the interaction between BA and antibiotics against MRSA, as well as their in vivo anti-infective efficacy, remain unexplored. Prior investigations conducted by our laboratory revealed that the minimum inhibitory concentration (MIC) of BA against the MRSA USA300 strain was 1250 μg/mL, whereas the MIC of oxacillin sodium (OXS) against the MRSA USA300 strain was recorded at 31.3 μg/mL, and gentamicin (GM) exhibited an MIC of 1.56 μg/mL against the same pathogen [23]. Based on the susceptibility criteria established by the National Committee for Clinical Laboratory Standards (NCCLS) in the United States, MRSA USA300 was classified as resistant to OXS but susceptible to GM [34]. In contrast, the MIC values for BA, OXS, and GM against the ATCC 310011 strain were determined to be 1250, 0.24, and 0.39 μg/mL, respectively, indicating that this strain is sensitive to both OXS and GM. Further investigations revealed that combining OXS with 1/4 MIC of BA reduced the OXS MIC against MRSA USA300 to 1/4 of its initial value, yielding a fractional inhibitory concentration index (FICI) of 0.5, which signifies a synergistic antibacterial interaction. Similarly, the combination of GM with 1/32 MIC of BA lowered the GM MIC to 1/4 of the original level, resulting in an FICI of 0.2815, thereby also demonstrating synergy. When 1/2 MIC of BA was used in conjunction with either OXS or GM against ATCC 310011, the MICs of these antibiotics were reduced to 1/8 and 1/32 of their initial levels, yielding FICI scores of 0.625 and 0.53, respectively, which indicates a partial synergistic interaction in terms of antibacterial efficacy [23]. Building upon these findings, the current research further investigates how the co-administration of BA and OXS influences both biofilm formation and virulence factors in MRSA USA300. For this purpose, GM was chosen as the positive control, while ATCC 310011 served as the reference strain. Furthermore, an in vivo mouse model of peritonitis triggered by MRSA USA300 was developed to assess the combined antibacterial and anti-inflammatory properties of BA and OXS. Experimental data demonstrated that the combination of BA with OXS, as well as with GM, exhibited favorable safety profiles in vitro. Specifically, no significant hemolysis of red blood cells was observed, with hemolysis rates remaining below 3% (as illustrated in Figure 1A–D). Unlike many conventional antibiotics that rely on singular modes of action—such as disrupting cell membranes, generating reactive oxygen species (ROS), or inhibiting biofilm development—the enhancement of these multifaceted mechanisms offers a promising strategy for improving therapeutic outcomes against bacterial infections [35]. Experiments designed to detect reactive oxygen species (ROS) revealed that co-administration of BA and OXS significantly enhanced intracellular ROS accumulation in MRSA USA300 and ATCC 310011, which was 1.2 and 2.4 times higher than that of the OXS-alone control group, respectively (Figure 1E,G). The possible explanation for this result is that OXS, as a β-lactam antibiotic, disrupts the integrity of the cell wall, thereby inducing overall metabolic disorders in bacteria and ultimately promoting the accumulation of intracellular ROS [36]. When combined with BA, it can enhance the permeability of the bacterial cell membrane, facilitate the entry of OXS into the cell, and reduce the ability of the bacteria to clear ROS, ultimately resulting in a significant accumulation of intracellular ROS [23]. Biofilm formation serves as a critical mechanism for bacterial resistance, primarily by establishing physical barriers that hinder antibiotic penetration and altering bacterial metabolism to diminish drug sensitivity [37,38]. Within these biofilms, bacteria are encased in an extracellular matrix predominantly consisting of proteins and extracellular polysaccharides (EPSs) [39]. EPSs create a multi-layered protective shield around the bacterial cells, which facilitates surface adhesion and offers substantial protection [40]. Using a phenol-concentrated sulfuric acid assay, it was determined that the combined application of BA and OXS effectively suppressed the synthesis of EPS components. The biofilm biomass associated with MRSA USA300 and ATCC 310011 exhibited reductions of 52.9% and 13.5%, respectively, relative to the levels observed with OXS monotherapy (Figure 2A,C). Subsequent investigations revealed that co-administration of BA and OXS significantly diminished the total protein content within the biofilms of MRSA USA300 and ATCC 310011, achieving decreases of 38% and 10% compared to treatment with OXS alone (Figure 2E,G). These findings suggest that the BA-OXS combination suppresses the production of EPS constituents and lowers overall biofilm protein levels, thereby demonstrating a synergistic effect against biofilm formation. This observation aligns closely with the work of Tsopmene et al., who reported similar synergistic anti-biofilm activities when thymol was combined with either cefixime or cefazolin [41].

Bacterial metabolic processes serve as a direct indicator of the biosynthesis of key components, including nucleic acids, proteins, and polysaccharides, while also reflecting the overall stability of the microbial community. These activities are fundamental to the establishment and structural integrity of biofilms [42]. Previous research has demonstrated that compromising the structural integrity of bacterial biofilms impedes material transport within the bacteria, thereby destabilizing their metabolic equilibrium [43]. MTT assay results revealed that, in contrast to the application of OXS alone, the combined treatment with BA and OXS led to a significant reduction in the metabolic activity of MRSA USA300 and ATCC 310011 biofilms, with decreases of 42% and 47.8%, respectively (Figure 2I,K). These findings suggest that the synergistic action of BA and OXS induces metabolic dysfunction in *Staphylococcus aureus* biofilm cells. The pathogenicity of *Staphylococcus aureus* is largely attributed to its virulence factors, which primarily include various enzymes and toxins. Specifically, lipases facilitate the hydrolysis of host lipids, which not only liberates fatty acids but also compromises the structural integrity of the host’s lipid components [44,45]. Meanwhile, staphyloxanthin, a toxin-related pigment, aids bacterial evasion of host immune responses by integrating into the bacterial cell membrane and neutralizing reactive oxygen species generated by immune cells [46,47]. In experiments assessing lipase activity via the measurement of transparent zone diameters, it was observed that the combined application of BA and OXS significantly decreased the size of these clear zones formed by MRSA USA300 and ATCC 310011 on LB agar plates supplemented with 1% tributyrin (Figure 3A,C). Furthermore, staphyloxanthin was extracted and quantified from MRSA USA300 and ATCC 310011 strains following treatment with BA and OXS. The findings indicated that, relative to the group treated with OXS alone, the staphyloxanthin levels in MRSA USA300 and ATCC 310011 subjected to the combined BA and OXS treatment were diminished by 38.9% and 18.2%, respectively (Figure 3E,G).

As a key regulatory element, the *sarA* gene directly binds to promoter regions to modulate the transcription of multiple virulence determinants, such as adhesins and toxic proteins [48,49]. Previous research has demonstrated that cinnamaldehyde can work in synergy with β-lactam antibiotics to suppress biofilm development by targeting the *sarA* regulator and diminishing its gene expression levels [50]. We employed quantitative PCR (qPCR) to examine how the combination of BA and OXS influences *sarA* expression in bacterial cells. Our data revealed that co-treatment with BA and OXS significantly reduced *sarA* transcription in MRSA USA300, resulting in a 30% decrease relative to the group receiving OXS monotherapy (Figure 3I). When BA and OXS were used in combination to treat ATCC 310011, *sarA* gene expression could also be downregulated, with an inhibition rate 32.2% higher than that of the group treated with OXS alone (Figure 3K). The presence of BA combined with OXS appears to suppress both biofilm development and the production of virulence determinants by downregulating *sarA* expression. Overall, the combination of baicalin and oxacillin sodium mainly exerts a synergistic effect of anti-toxicity and anti-biofilm through the accumulation of ROS and the downregulation of *sarA.* However, other phytochemicals combined with antibiotics also have other antibacterial targets in bacteria. For example, Manuka honey targets mecR1 to reverse oxacillin resistance [31]. It is worth noting that this combined antibacterial regimen exhibits strain-specific effects. It shows stronger efficacy in terms of ROS accumulation and bacterial metabolic activity inhibition against ATCC 310011, but its effect in inhibiting biofilm formation is more prominent in MRSA USA300, reflecting inherent biological differences in the oxidative stress tolerance and biofilm regulatory pathways of the two strains. Further validation of the general applicability of this combined strategy is needed by including more clinical MRSA isolates in the future. Subsequently, we developed a murine peritonitis model using MRSA USA300. Our results indicated that the combined administration of BA and OXS partially mitigated weight loss in infected mice relative to the untreated MRSA USA300 cohort (Figure 4A). Furthermore, this therapy significantly lowered both the count of inflammatory cells and the concentrations of inflammatory markers in the plasma. Specifically, when compared to the MRSA USA300 group, the combined treatment group exhibited reductions exceeding 50% in plasma levels of white blood cells, lymphocytes, monocytes, and neutrophils. Additionally, the expression of pro-inflammatory cytokines TNF-α, IL-6, and IL-1β was reduced by 10%, 8%, and 27%, respectively (Figure 4B–D and Figure 5B–D). Moreover, in comparison to either the infection-only group or those receiving monotherapy, the concurrent administration of BA and OXS led to a marked decline in bacterial burdens within the cardiac, hepatic, splenic, pulmonary, and renal tissues of mice suffering from peritonitis (Figure 5A). Furthermore, this combined treatment effectively mitigated histological damage in the liver, kidneys, and peritoneal cavity (Figure 6A). Our investigation further revealed that the synergistic application of BA and OXS substantially ameliorated oxidative stress injuries induced by MRSA USA300 in these animals. This protective effect was evidenced by elevated plasma concentrations of superoxide dismutase (SOD) and catalase (CAT), alongside reduced levels of malondialdehyde (MDA) (Figure 6B–D). Ultimately, ELISA assays demonstrated that, relative to the group infected with MRSA USA300 alone, the combined BA and OXS intervention significantly lowered the expression of key markers for hepatic and renal function—specifically aspartate aminotransferase (AST), alanine aminotransferase (ALT), creatinine (Cr), and blood urea nitrogen (BUN). These indicators reverted to levels comparable to those of the control group, suggesting that the co-administration of BA and OXS exhibits a favorable safety profile with no discernible toxic impact on liver or kidney functions (Figure 6E–H). In conclusion, the combined use of BA and OXS exerts anti-biofilm and anti-virulence activities and can exert a positive therapeutic effect on mice with peritonitis induced by MRSA USA300. These findings align with prior research indicating that synergistic applications of botanical extracts and antimicrobial agents possess potent anti-infective capabilities in living organisms. For instance, Wang et al. reported that Rhein successfully reinstated colistin sensitivity in multidrug-resistant *Escherichia coli* strains harboring the mcr-1 gene, both in laboratory settings and within animal subjects. In experiments involving peritonitis, the joint use of Rhein and colistin markedly decreased bacterial burdens in various organs, suppressed severe inflammatory responses, and exhibited negligible organ toxicity [51]. Consequently, pairing plant-derived compounds with antibiotics shows considerable promise for addressing infections caused by drug-resistant MRSA. Nevertheless, this investigation is not without its constraints. Specifically, static in vitro biofilm results cannot fully recapitulate the complex in vivo infection microenvironment. To date, there has been no direct in situ verification of the link between biofilm formation and the suppression of virulence in the context of abdominal infections. Consequently, it remains challenging to clearly delineate the respective roles played by the drug’s inherent antibacterial properties and the modulation of the host’s immune response. Future investigations will prioritize the examination of abdominal lesions in their native state, aiming to bridge the gap between molecular phenotypes observed in vitro and therapeutic outcomes in vivo. By integrating both cellular and animal model systems, we intend to disentangle and quantify the individual contributions of these dual mechanisms. Furthermore, this study has not yet elucidated the detailed molecular pathways between baicalin and the upstream and downstream regulation of *sarA* and ROS production. In the future, we plan to use ROS-specific inhibitors for intervention and transcriptome sequencing to analyze the molecular targets of baicalin in regulating this pathway. In addition, several potential confounding factors remain in this animal model. Variations in individual immune background, sex-related biological characteristics of mice, and fluctuations in bacterial inoculum dose may affect in vivo bacterial clearance and disease progression, thereby interfering with the evaluation of drug efficacy. In follow-up studies, we will strictly control the baseline immune status and sex of animals and standardize the bacterial inoculation dose to reduce bias caused by confounding variables.

## 5. Conclusions

From a clinical perspective, the substantial prevalence and fatality rates associated with Methicillin-resistant *Staphylococcus aureus* (MRSA) infections constitute a severe hazard to public health. Consequently, synergistic therapies involving plant-derived extracts alongside conventional antibiotics have gained prominence as novel therapeutic strategies for combating MRSA. Our investigation revealed that co-administering baicalin with oxacillin sodium exhibits favorable biosafety profiles. This combination facilitates the intracellular generation of reactive oxygen species (ROS) within MRSA USA300 strains. Furthermore, it demonstrates potent anti-biofilm efficacy by suppressing the production of extracellular polymeric substance (EPS) constituents, reducing total protein levels, and diminishing the metabolic activity of biofilm cells. Additionally, our results indicate that this drug pairing downregulates the *sarA* gene expression, which in turn curtails the biosynthesis of key virulence factors, including lipase and staphyloxanthin. Therefore, this study proposes that ROS accumulation triggered by the baicalin-oxacillin sodium combination constitutes the major driver of bactericidal activity and biofilm disruption. Combined inhibition of *sarA* expression predominantly mediates the attenuation of bacterial virulence. Crosstalk between these two pathways synergistically mediates the overall inhibitory effect of this combinatorial regimen against MRSA USA300. In murine models of peritonitis triggered by MRSA USA300, the combined treatment displayed marked antibacterial and anti-inflammatory properties, effectively mitigating pathological injuries within the abdominal cavity, while also mitigating oxidative stress-induced injury within the organism. Consequently, this results in protective benefits for mice suffering from peritonitis caused by MRSA USA300. This study demonstrates that baicalin combined with oxacillin sodium has the potential to be developed as an adjuvant therapeutic strategy for MRSA intra-abdominal infection, with promising translational prospects.

## Data Availability

The original contributions presented in this study are included in the article. Further inquiries can be directed to the corresponding authors.

## Abbreviations

BA	baicalin
DMSO	dimethyl sulfoxide
EDTA	Ethylene Diamine Tetraacetic Acid
EPS	extracellular polymeric substance
FICI	fractional inhibitory concentration index
GM	gentamicin
HE	hematoxylin eosin
MRSA	methicillin-resistant *Staphylococcus aureus*
MTT	3-[4,5-dimethylthiazol-2-yl]-2,5-diphenyltetrazolium bromide
OXS	Oxacillin sodium
PBS	phosphate-buffered saline
qPCR	quantitative real-time polymerase chain reaction
SD	standard deviation
VAN	vancomycin
